# Analysis of *PRM1* and *PRM2* Polymorphisms in Iranian Infertile Men with Idiopathic Teratozoospermia

**DOI:** 10.22074/ijfs.2019.5650

**Published:** 2019-01-06

**Authors:** Fatemeh Dehghanpour, Farzaneh Fesahat, Seyed Mohsen Miresmaeili, Ehsan Zare Mehrjardi, Ahmad Honarju, Ali Reza Talebi

**Affiliations:** 1 Research and Clinical Center for Infertility, Yazd Reproductive Sciences Institute, Shahid Sadoughi University of Medical Sciences, Yazd, Iran; 2 Medical Biotechnology Research Center, Ashkezar Branch, Islamic Azad University, Yazd, Iran; 3 Reproductive Immunology Research Center, Shahid Sadoughi University of Medical Sciences, Yazd, Iran; 4 Department of Biology, Science and Arts University, Yazd, Iran

**Keywords:** Single Nucleotide Polymorphisms, Sperm, Teratozoospermia

## Abstract

Single nucleotide polymorphisms (SNPs) in a number of genes involved in sperm maturation are considered as one of the main factors for male infertility. The aim of the present case-control study was to examine the association of SNPs in protamine1 (*PRM1*) and protamine2 (*PRM2*) genes with idiopathic teratozoospermia. In this case-control study, some SNPs in PRM1 (c.49 C>T, c.102 G>T and c.230A>C) and *PRM2* (rs545828790, rs115686767, rs201933708, rs2070923 and rs1646022) were investigated in 30 idiopathic infertile men with teratozoospermia (case group) in comparison with 35 fertile men (controls). Genotyping of SNPs was undertaken using polymerase chain reaction (PCR)-direct sequencing. For *PRM1*, c.230A>C, as a synonymous polymorphism, was detected in both teratozoo- spermic men (heterozygous n=26, homozygous minor n=1) allele frequency C(48) A(52) and controls (heterozygous n=15, homozygous minor n=4). All cases and controls were genotyped for rs545828790 in *PRM2*, a missense poly- morphism, as well as rs115686767 and rs201933708, both of which synonymous variants. The findings showed an intronic variant in *PRM2* (rs2070923) was also present in both groups. Also, rs1646022, a missense polymorphism, occurred in teratozoospermic men (heterozygous n=10, homozygous minor n=5) and controls (heterozygous n=13, homozygous minor n=2). However, there were no significant differences in SNPs of *PRM1* and *PRM2* between the two groups, however, for c.230A>C, the frequency of the CA genotype was significantly higher in infertile men with teratozoospermia (P=0.001). We demonstrate that *PRM2* G398C and A473C polymorphisms were associated with the teratozoospermia and its genetic variation was in relation to semen quality, sperm apoptosis, and morphology in the Iranian population. This study is a preliminary study and presenting data as part of a future comprehensive study to clinically establish whether these gene polymorphisms are biomarkers for susceptibility to teratozoospermia.

Genetic factors are responsible for 50% or more of male infertility etiology and nearly 7% of men suffer from infertility worldwide (1, 2). It is generally accepted that abnormalities in sperm chromatin and DNA are one of the main factors affecting pregnancy rates. Sperm DNA packaging, which occurs during spermiogenesis, is a unique process because of histone-protamine replacement. 

Two types of protamines are known to exist in humans, amely protamine1 (PRM1) and protamine 2 (PRM2). The expression of this two proteins in the sperm nucleus is almost equal (3). The protamine proteins are characterized by an arginine-rich core and cysteine residues (4). Protamine has specialized in sperm for several reasons including more chromatin condensation, faster spermatozoa, effective oocyte fertilization, protecting the maternal genome from nuclease and toxins, permitting oocyte to reprogramme the paternal genome, and for having an imprinting pattern and reactivation upon fertilization (5).

Therefore, due to the importance of protamines in male fertility, many studies have shown altered expression of protamines in several groups of infertile men (3, 6-8). *PRM1* and *PRM2* are located on chromosome 16 and both genes contain a single intron (9). A number of reports have verified different variations in *PRM1* and *PRM2* sequences (NM_002761.2 and NM_002762.3) in humans with various associations with male infertility (10-13). Infertile men with high levels of abnormal sperm morphology are considered to teratozoospermia (14). There is some evidence that protamine mutations or polymorphisms may induce conformational changes the protein level, altering their incorporation into sperm chromatin thus leading to sperm defects. *PRM* deficiency causes sperm morphology defects, motility reduction, and infertility as a result of haploinsufficiency in mice (15, 16). *PRM1* variant rs35576928 is a single nucleotide polymorphism (SNP) that is present at a significantly higher frequency in infertile patients with non-obstructive azoospermia and altered morphology of the spermatozoa (17). Also, variants of *PRM1* and *PRM2* have been shown to be associated with male infertility and abnormal sperm morphology. A polymorphism in the *PRM1* promoter (190 C.A) is known to increase *PRM1* to *PRM2* ratio (18-20). The aim of our study was to examine the association of SNPs in *PRM1* and *PRM2* with idiopathic teratozoospermia.

In this case-control study, semen samples were collected from 35 fertile men (control group) and 30 infertile men (case group) referred to our Andrology Lab at the Research and Clinical Center for Infertility of Yazd. Sperm samples were obtained after informed consent from the participants. A comprehensive evaluation was undertaken to identify the etiology of infertility including physical examination, smoking history, and reproductive hormonal assays. The infertile man was defined as a man who had no child after a period of unprotected intercourse for more than one year. The control group included fertile donors with one naturally conceived child during the past 12 months who also had normal semen parameters according to the recommendations of the World Health Organization (WHO, 2010). Heavy smokers (more than one pack of cigarettes per day during the past year), drug addicts, alcohol consumers, men with a history of varicocele and those aged more than 45 years were excluded from the study. Theliquefiedsemen of each man was evaluated for sperm parameters according to to WHO 2010 (14). Sperm morphology was evaluated using the strict criteria of Kruger et al. (21) and at least 200 cells were examined per slide. To determine the genetic status of protamine genes, the DNA sample was extracted using the salting out method from the peripheral blood of each individual (22). This study was approved by the Ethics Committee of the Yazd Research and Clinical Center for Infertility.

Genomic DNA isolation from peripheral blood samples was performed using the protease and phenol purification protocol (23). Each polymerase chain reaction (PCR) reaction consisted of 1-2 µl (100 ng) of DNA, 0.5-1 µl (0.2 µM) of each specified primer (Pishgam co., Iran) (Table 1) (11) and 12.5 µL of the 2X mastermix (amplicon) in a total volume 25 µL. The cycling conditions for PCR were an initial denaturation of DNA at 94°C for 5 minutes, 35 cycles of 94°C for 30 seconds, 66°C for *PRM1* and 69°C for *PRM2* for 45 seconds, 72°C for 30 seconds, and a final extension of 10 minutes at 72°C. To verify fragment lengths, 2 µl of each PCR fragment was electrophoresed on a 1.5% agarose gel stained with SYBR DNA Safe Stain (Invitrogen, USA).

**Table 1:** Two primer pairs for amplification of the *PRM1* and *PRM2* genes


Gene	Sequence primer (5'-3')	Product size (bp)

*PRM1*	F: cccctggcatctataacaggccgc	558
	R: tcaagaacaaggagagaagagtgg	
*PRM2*	F: ctccagggcccactgcagcctcag	599
	R: gaattgctatggcctcacttggtg	


After PCR, all of the PCR products were purified and sequenced on an Applied Biosystems 3730 XL DNA analyzer according to the manufacturer’s instructions. Using designed primers (forward and reverse), the amplified products with sizes of 557 nucleotides (from-42 to 515) for *PRM1* and 599 nucleotides (from 49 to 648) for *PRM2* were sequenced. Chromatograms were 3 analyzed using Chromas 2 (Technelysium Pty. Ltd., South Brisbane, QLD, Australia).

In this study, we used SPSS 20 (SPSS Inc., Chicago, IL, USA) for all statistical analyses. The frequency of SNPs in *PRM1* and *PRM2* in case and control groups were compared using logistic regression. Differences between groups were examined using one-way ANOVA (followed by Turkey test) for sperm characteristics.

A total of 65 semen samples were examined in two groups. The mean age of participants was 35.21 ± 5.5 vs. 33.71 ± 4.5 years in the case and control groups respectively. In the case and control group, we observed three SNPs in *PRM1* and five SNPs in *PRM2*, namely C230A, G102T, and C49T in *PRM1*, and C288T, C401T, C248T, G398C, A473C, and G271C in *PRM2*. The PCR products were verified on agarose gels (Fig .1).

The frequency of these SNPs differed in groups of fertile and infertile men. The three SNPs G102T and C49T in *PRM1*, and C248T in *PRM2* were not observed in either group. Other SNPs were found in non-coding regions (Table 2).

Table 3 shows the association of the most frequent genotypes (three SNPs) with seminal characteristics of participants (Table 3).

Abnormal morphology, as well as sperm apoptosis (TUNEL+), was significantly elevated in the GG genotype compared with other genotypes in rs1646022 and rs2070923 in *PRM2*. However, in *PRM1* rs737008, the highest percentage of abnormal morphology, apoptotic sperms, and abnormal motility belonged to the AA genotype. There was no difference between genotypes regarding sperm protamine deficiency and sperm concentrations for all SNPs in *PRM1* and *PRM2* (Table 4).

In this study, infertile men with a history of defects at sperm head morphology and stretch of this region (tapered head) were analyzed for *PRM1* and *PRM2* polymorphisms and compared with fertile men. The findings showed an intronic variant in *PRM2* (rs2070923) which was also present in both groups. Also, rs1646022, a missense polymorphism, occurred in teratozoospermic men (heterozygous n=10, homozygous minor n=5) and controls (heterozygous n=13, homozygous minor n=2). However, there were no significant differences in SNPs of *PRM1* and *PRM2* between the two groups for c.230A>C, the frequency of the CA genotype was significantly higher in infertile men with teratozoospermia.

**Fig.1:**
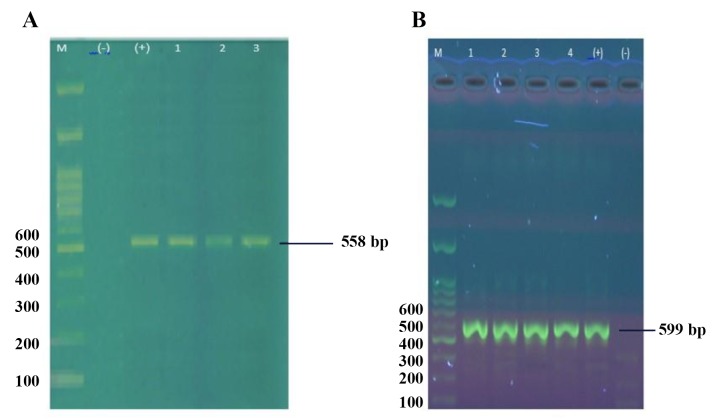
Polymerase chain reaction (PCR) product of genomic DNA using specific primers. A. *PRM1* with 557 bp and B. *PRM2* with 599 bp length was detected. M; Molecular marker (100 bp), (+); Positive control, and (-); Negative control.

**Table 2:** The frequency of single nucleotide polymorphisms (SNPs) in PRM1 and PRM2 in case and control groups


Gene	Nucleotide change	Region	AA change	NCBI ID	Infertile		Fertile		P value
					Genotypefrequency (%)	Allele frequency	Genotypefrequency (%)	Allele frquency		

P1	C230A	Exon	None	rs737008	CC: 0.0	C(48)	CC: 26	C(54.5)	0.000
					CA: 96	A(52)	CA: 57	A(45.5)	0.002
					AA: 4		AA: 17		
	G102T	Exon	R→S	-	GG: 100	G(100)	GG: 100	G(100)	NS
					GT: 0.0	T(0)	GT: 0.0	T(0)	NS
					TT: 0.0		TT: 0.0		
	C49T	Exon	R→C	-	CC: 100	C(100)	CC: 100	C(100)	NS
					CT: 0.0	T(0)	CT: 0.0	T(0)	NS
					TT: 0.0		TT: 0.0		
P2	C288T	Intron	None Coding	rs115686767	CC: 100	C(100)	CC: 94.11	C(97.05)	NS
					CT: 0.0	T(0)	CT: 5.89	T(2.95)	NS
					TT: 0.0		TT: 0.0		
	C401T	Intron	None Coding	rs545828790	CC: 100	C(100)	CC: 100	C(100)	NS
					CT: 0.0	T(0)	CT: 0.0	T(0)	NS
					TT: 0.0		TT: 0.0		
	C248T	Exon	E-Q	-	CC: 100	C(100)	CC: 100	C(100)	NS
					CT: 0.0	T(0)	CT: 0.0	T(0)	NS
					TT: 0.0		TT: 0.0		
	G398C	Intron	None Coding	rs1646022	GG: 44.44	G(63.87)	GG: 31.25	G(62.5)	0.004
					GC: 38.86	C(36.13)	GC: 62.5	C(37.5)	0.012
					CC: 16.7		CC: 6.25		
	A473C	Intron	None Coding	rs2070923	AA: 33.33	A(50)	AA: 43.75	A(63.55)	0.073
					AC: 33.34	C(50)	AC: 39.59	C(36.45)	0.007
					CC: 33.33		CC: 16.66		
	G 271C	Intron	None Coding	rs201933708	GG: 100	G(100)	GG: 93.75	G(96.87)	NS
					GC: 0.0	C(0)	GC: 6.25	C(3.13)	NS
					CC: 0.0		CC: 0.0		


Logistic Regression Modeling was used for statistical analysis. All P values were two-sided and considered significant at the 0.05 level and showed the comparisons between the allele frequencies in case and control groups. P1; PRM1, P2; PRM2, AA; Amino acid R; Arginine, S; Serine, C; Cysteine, Q; Termination codon, E; Glutamic acid , and NS; No significant.

**Table 3:** Association of C230A polymorphism in PRM1 with sperm characteristics


Genotype	Apoptosis	Protamine deficiency	Abnormal motility	Concentration	Abnormal morphology

CC (n=6)	45.66 ± 16.23	35.6 ± 12.34	41.85 ± 6.89	64.83 ± 58.67	5.26 ± 3.49
CA (n=55)	30.8 ± 12.45	27.33 ± 9.67	45.33 ± 7.34	77.47 ± 60.5	48.48 ± 29.78
AA (n=4)	56 ± 18.96	29.66 ± 10.56	23.5 ± 3.45	49.25 ± 32.75	1.4 ± 0.32
P value	<0.001^*^	0.157	<0.001^*^	0.602	<0.001^*^


Values are presented as a mean ± standard deviation. Tukey’s test was used for statistical analysis. *; The P<0.05 were considered to indicate statistical significance. Evaluating the sperm parameters was according to the World Health Organization (WHO, 2010).

**Table 4:** Association of PRM2 G398C and A473C polymorphisms with human sperm characteristics


Genotype	Apoptosis	Protamine deficiency	Abnormal motility	Concentration	Abnormal morphology

rs1646022					
GG (n=6)	43.4 ± 18.3	28.75 ± 9.25	47 ± 8.45	74.4 ± 34.5	2.33 ± 2.13
GC (n=55)	29.9 ± 9.94	30.9 ± 15.45	57.54 ± 12.36	52.54 ± 28.75	28.7 ± 18.5
CC (n=4)	12.25 ± 3.88	37.75 ± 17.72	60 ± 14.87	74.5 ± 38.65	27.75
P value	<0.001^*^	0.63	0.12	0.11	0.003^*^
rs2070923					
AA (n=16)	24.83 ± 8.25	27.33 ± 6.85	55.66 ± 10.76	84.83 ± 64.23	33.33 ± 23.32
AC (n=34)	13.5 ± 3.97	26 ± 11.83	63.33 ± 8.69	102.66 ± 85.43	37 ± 30.54
CC (n=15)	41 ± 14.28	26.66 ± 12.35	44.66 ± 10.73	54.66 ± 47.23	5 ± 4.55
P value	<0.001^*^	0.92	0.02^*^	0.12	<0.001^*^


*; The P<0.05 were considered to indicate statistical significance. Data are presented as mean ± SD. Post-hoc test to ANOVA was used for statistics evaluating the sperm parameters was according to the World Health Organization (WHO, 2010).

Protection and support of the sperm genome are the main functions of protamines. It is shown that incomplete protamination of sperm DNA causes high susceptibility of the genome to nucleases, endogenous and exogenous free radicals and mutagens (24). Studies have also DNA damage (25, 26). Consistent with our results, Aoki et al. (27) analyzed 15 SNPs such as G102T and C203A in *PRM1* and observed similar frequencies between an infertile population and normal controls. But, in contrast to Aoki et al. (27), C203A(rs737008) was different between two groups in the present study. The frequency of C49T, located in the exonic region, was the same between groups whereas these results were different in study by Jodar et al. (28). They found that infertile men with normal sperm count but with both abnormal motility and morphology had a nonsynonymous substitution at position C49T (R17C). A recent review article demonstrated that C230A variant had a higher frequency infertile men compared with controls (19) which were also in agreement with our results. In addition, Tanaka et al. (11) reported 5 gene polymorphisms in *PRM1* and 3 polymorphisms in *PRM2* in infertile men. Cho et al. (15) found one stop-gained variant, which converted glutamic acid to a stop codon (known as C248T), in one individual from an infertile azoospermic group. We identified G398C (rs1646022) and A473C (rs2070923) in our samples, however, Tanaka et al. (11) did not show the above-mentioned SNPs in azoospermic and oligospermic patients. Another study conducted by Jiang al. (19) showed that G398C is present in infertile men, which was in line with our findings.

Although Aoki et al. (27) identified15 SNPs, however, given that their frequencies in cases were almost identical to controls, they did not consider them as the underlying genetic cause of abnormalities in the expression of PRMs in infertile couples. In this study, the C281T substitution was selected and evaluated among other SNPs and as in Aoki et al. (27) results, we did not observe C281T in either group. It is likely that the variation in results of these studies is due to differences in study populations, which in fact shows that most of these SNPs were at variable frequencies in different populations, indicating that the distribution of genotypes related to different polymorphisms of *PRM1* and *PRM2* genes have ethnic variation. We also detected significant associations between the frequencies of GG and CC genotypes of *PRM2* rs1646022 and rs2070923 respectively with apoptosis, morphology and total motility. Interestingly, these genotypes were the most frequent genotypes in infertile men with taper head spermatozoa. In contrast, there was no association between genotype AA at rs737008 with male infertility. In our previous study, we reported sperm protamine deficiency, lower rates of normal sperm parameters, and apoptosis in infertile men with idiopathic teratozoospermia compared to the controls.

We saw that the concentration of sperm cells was lower in the case group than controls. Also, total motility and sperm morphology were significantly lower in the case group than in the control group. Furthermore, we showed significantly higher rates of protamine deficiency as well as sperm apoptosis in patients with tapered sperm compared with the fertile group using CMA3 florescent staining and TUNEL assay respectively (29). The findings of the present survey are in agreement with the mentioned previous study, demonstrating the probable relationship between male infertility in patients with tapered head sperms and *PRM2* polymorphisms as well as sperm protamine deficiency, apoptosis rate, and morphology. These damages may affect the quality of the ejaculated spermatozoa and decrease their fertility potential in natural conception or ART cycles. Furthermore, a recent study reported that *PRM2* G398C is associated with the pathogenesis of male infertility in idiopathic infertile men from Chinese Han population, which was in line with our results despite having studied different populations (18). Also, another recent study reported that the c.-190 C>A transversion may be involved in the susceptibility for oligozoospermia and could be used as a non-invasive molecular marker for genetic diagnosis of idiopathic oligozoospermia (30). Finally, a more recent study showed that c.-9C>T and c.368A>G polymorphisms of H2BFWT may be genetic risk factors for male infertility so we suggested these polymorphisms investigated in the teratospermia group (31).

We show that *PRM2* G398C and A473C polymorphisms are associated with male infertility in men with teratozoospermia and sperm parameters including semen quality, sperm apoptosis, and morphology in the Iranian population. This study is a preliminary study of a larger comprehensive research program aiming to identify clinically relevant polymorphisms as biomarkers for susceptibility to teratozoospermia.
